# Supplementary material for: Paracrine Bone-Derived Senescent Secretome Induces Spatially Patterned ECM and Biomechanical Vulnerability in Human Brain Organoids
Source: bioRxiv. 2025 Aug 12:2025.08.11.669674. Preprint. [Version 1] doi: 10.1101/2025.08.11.669674 (PMC12363841; doi:10.1101/2025.08.11.669674)
Supplement: Supplement 1 [file media-1.pdf]

Paracrine Bone-Derived Senescent Secretome Induces Spatially Patterned ECM and Biomechanical Vulnerability in Human Brain Organoids

Chanul Kim [1], Luke Daniel Ofria [2], Anannya Kshirsagar [2], Elizabeth Gomez Flores [1], Sai Kulkarni [2], Alan Y. Liu [3], James L. Kirkland [4], Annie Kathuria [2*], Maryam Tilton [5*]

[1] Department of Biomedical Engineering, The University of Texas at Austin, Austin, TX, USA

[2] Department of Biomedical Engineering, Johns Hopkins University, Baltimore, MD, USA

[3] Optics11 Life Inc., Boston, Massachusetts, USA

[4] Center for Advanced Gerotherapeutics, Division of Endocrinology, Department of Medicine, Cedars-Sinai Medical Center, Los Angeles, CA, USA

[5] Walker Department of Mechanical Engineering, The University of Texas at Austin, Austin, TX, USA

*Corresponding authors:

M. Tilton: maryam.tilton@austin.utexas.edu and A. Kathuria: annie_kathuria@jhu.edu

Extended Data

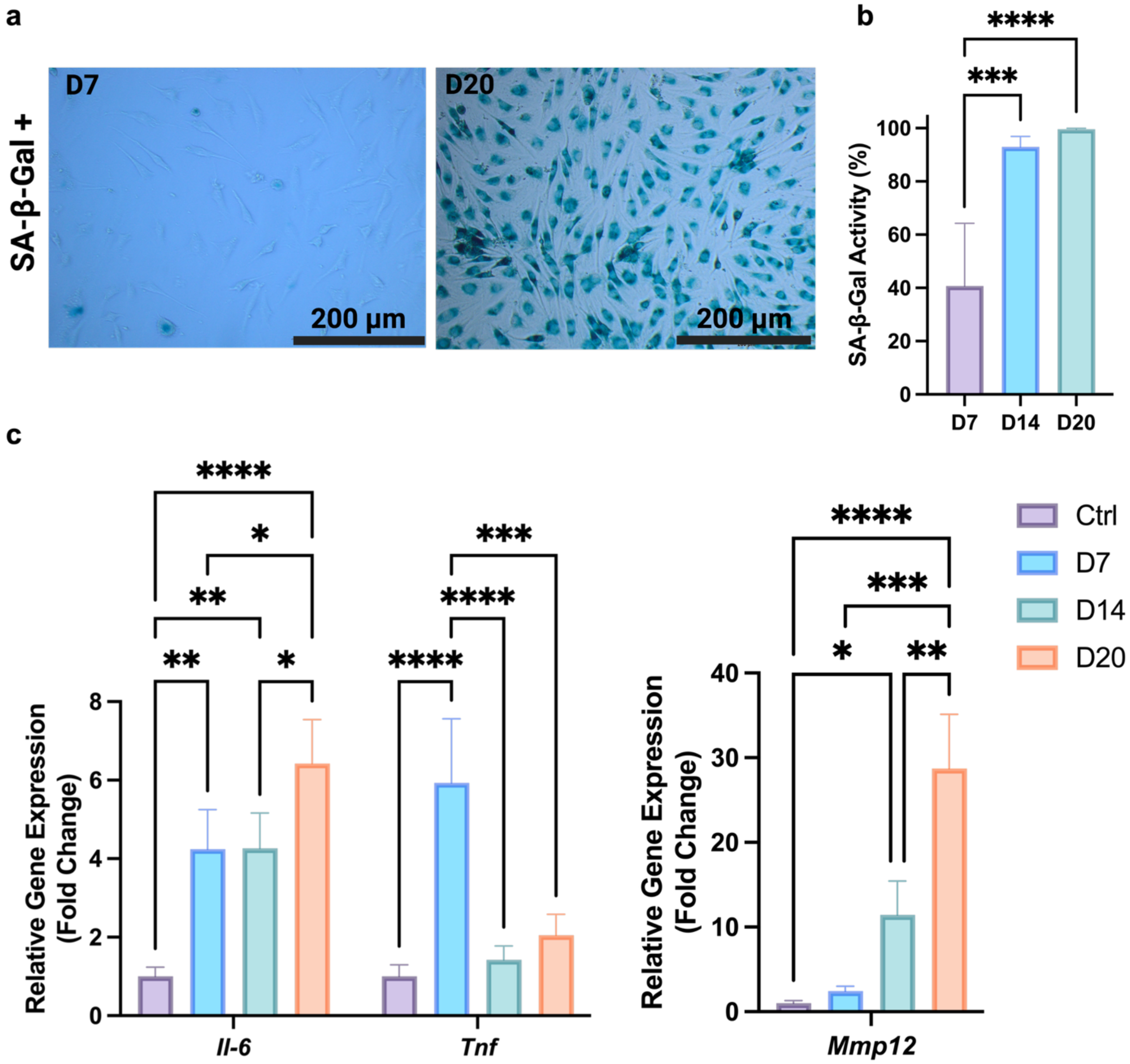

Extended Data Fig. 1| Validation of senescent phenotype and SASP production in irradiated osteocyte cultures used for SASP-CM generation. a, Representative images of SA-β-Gal staining osteocytes cultures post-irradiation on days 7 and 20. **b,** Quantification of SA-β-Gal + cells as a percentage of total cells. **c,** RT-qPCR analysis of SASP gene expression (*Il-6, Tnf-α, Mmp12*) in irradiated versus control non-senescent cultures on day 20, normalized to Gapdh (Housekeeping gene) and shown as fold change relative to control. Data are presented as mean ± SD for N=3 biological replicates. Significance levels are denoted as *: $p < 0.05$, **: $p < 0.01$, ***: $p < 0.001$. ****: $p < 0.0001$. **All analyses were performed on the same batch of irradiated cultures used for SASP-CM generation in this work.** Panels **a-c** are reproduced

from Tilton et al., *Small* 2025, with permission, to validate the preparation of SASP-CM used in the present study.

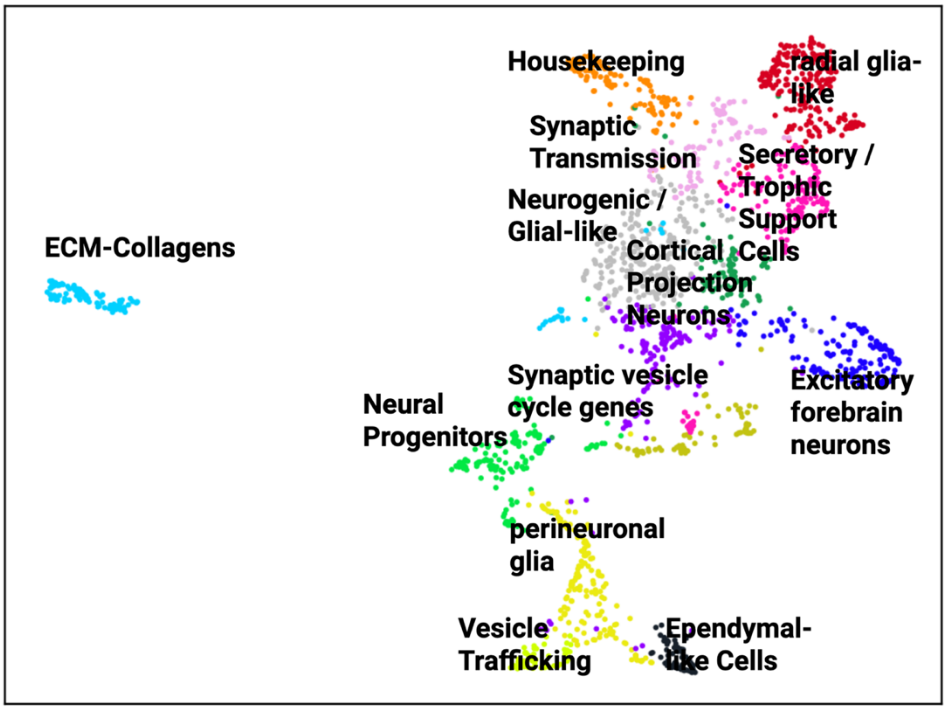

Extended Data Fig. 2| Single-nucleus transcriptomic profiling of 2-month-old cerebral organoids reveals diverse neuronal and non-neuronal populations. UMAP visualization of single-nucleus RNA-seq data from 2-month-old cerebral organoids identifies 14 transcriptionally distinct clusters, each annotated by enriched gene signatures. Annotated populations include excitatory forebrain neurons (e.g., *GRIA2* $^{+}$, *RBFOX3* $^{+}$), synaptic vesicle cycle-enriched neurons (*SNAP25* $^{+}$, *PCLO* $^{+}$), cortical projection neurons (*NRXN1* $^{+}$, *TBR1* $^{+}$), and multiple non-neuronal subtypes such as neurogenic/glial-like cells (*NEAT1* $^{+}$, *RMST* $^{+}$), radial glia-like progenitors (*MIAT* $^{+}$, *MBD5* $^{+}$), and vesicle trafficking-associated trophic support cells (*VEGFA* $^{+}$, *TRPM7* $^{+}$). ECM clusters (*COL1A1* $^{+}$, *PRRX1* $^{+}$) and ECM-producing cells are also detected, alongside a small population of motile ciliated (ependymal-like) cells. This transcriptional landscape demonstrates advanced neuronal subtype emergence and, characteristic of developing cerebral organoid at this stage.

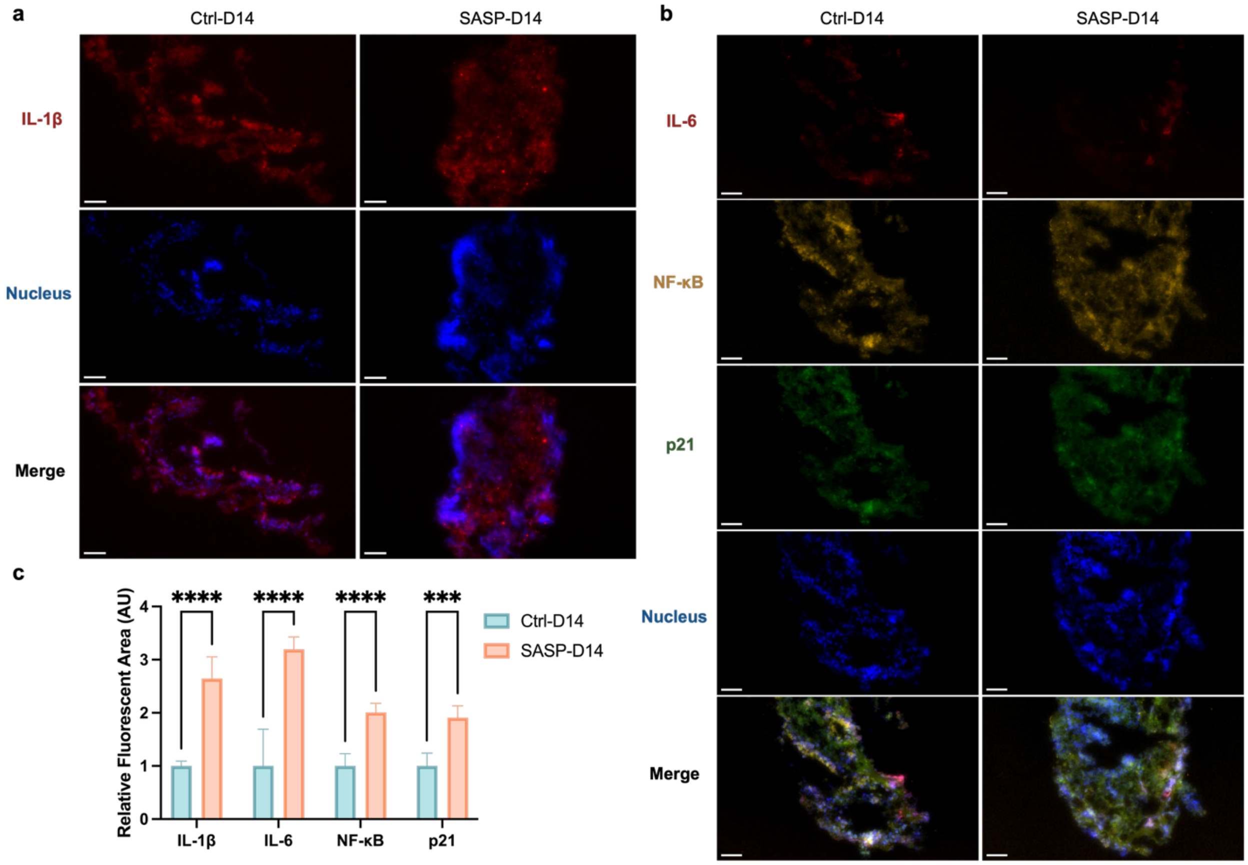

Extended Data Fig. 3| Immunofluorescence analysis of cerebral organoids following SASP-CM treatment. a, Representative immunofluorescence staining of cerebral organoids showing IL-1β (red) and nuclei (blue, DAPI). **b,** Representative multiplex immunofluorescence staining of cerebral organoids showing IL-6 (far-red), NF-κB (yellow), p21 (green), and nuclei (blue, DAPI). **c,** Quantification of fluorescence area for IL-1β, IL-6, NF-κB, and p21. Fluorescent regions were measured using Fiji (N=5). Values were normalized to the mean area of the Ctrl-D14 group for each marker. Data are shown as mean ± SD. Significance levels are denoted as ***: $p < 0.001$, ****: $p < 0.0001$. All Images in the figure were acquired using a 20× objective lens with a scale bar: 50μm
